# Identification of inflammatory-related gene signatures to predict prognosis of endometrial carcinoma

**DOI:** 10.1186/s12863-022-01088-0

**Published:** 2022-10-07

**Authors:** Linlin Chen, Guang Zhu, Yanbo Liu, Yupei Shao, Bing Pan, Jianhong Zheng

**Affiliations:** Tongde Hospital of Zhejiang Province, Hangzhou, 310012 China

**Keywords:** Prognosis, Endometrial carcinoma, TCGA, Inflammation-related

## Abstract

Little is known about the prognostic risk factors of endometrial cancer. Therefore, finding effective prognostic factors of endometrial cancer is the vital for clinical theranostic. In this study, we constructed an inflammatory-related risk assessment model based on TCGA database to predict prognosis of endometrial cancer. We screened inflammatory genes by differential expression and prognostic correlation, and constructed a prognostic model using LASSO regression analysis. We fully utilized bioinformatics tools, including ROC curve, Kaplan–Meier analysis, univariate and multivariate Cox regression analysis and in vitro experiments to verify the accuracy of the prognostic model. Finally, we further analyzed the characteristics of tumor microenvironment and drug sensitivity of these inflammatory genes. The higher the score of the endometrial cancer risk model we constructed, the worse the prognosis, which can effectively provide decision-making help for clinical endometrial diagnosis and treatment.

## Introduction

Less is still known about endometrial cancer, the most common gynecological cancer in developed nations [1–3]. There are 140,000 new cases of endometrial cancer worldwide each year, accounting for approximately 6% of new cancer cases and 3% of cancer deaths each year [4, 5]. Endometrial cancer, a complex gynecological neoplasm, is classified into type I (80–90%) and type II (10- 20%) based on clinical, endocrine and epidemiological features [6]. Currently, total hysterectomy, pelvic and para-aortic lymphadenectomy and bilateral salpingo-oophorectomy are the standard surgical treatments for endometrial cancer [7]. Most patients with endometrial cancer in the early stage have a better outcome after surgical resection. Adjuvant therapy, including radiation therapy, vaginal brachytherapy and chemotherapy, is available for women with advanced pathologic stage [8]. Studies have shown that postoperative recurrence is a major cause of increased mortality in endometrial cancer [9, 10]. Although traditional clinical features including tumor grade, FIGO staging, histological type, lymph node metastasis and myometrial infiltration are currently considered as risk factors to be associated with the prognosis of endometrial cancer [11], while they cannot precisely predict the prognosis of endometrial cancer. Therefore, finding the optimal predictive prognostic factors for endometrial cancer is the key of clinical research [12].

Solid tumors, including endometrial cancer, consist of nonmalignant mesenchymal cells, neoplastic cells and migratory hematopoietic cells [13]. Complex interactions between different cell types in the tumor microenvironment can impact the cancer growth, progression, metastasis and angiogenesis [12]. Inflammatory cells and inflammatory mediators are the main components of the tumor microenvironment. Inflammation is a key process in tumor-associated disease [13, 14]. In certain sources of cancer, inflammatory conditions precede malignancy development, while in others, the inflammatory environment that promotes tumors is driven by oncogenic changes. The prognosis of patients is related to the many clinical manifestations of tumor-associated inflammation. The recurrence and mortality in patients undergoing curative resection for cancer could be reduced after perioperative use of non‐steroidal anti‐inflammaory drug (NSAID) [15]. Studies have shown a 40% reduction in both recurrence and mortality rates in patients who used NSAID during the time of undergoing curative resection with rectal cancer [16, 17].

The role of inflammation in endometrial cancer development is well known [18, 19]. Endometrial cancer is immunogenic and is associated with a response to PD-1/PD-L1 inhibitors, resulting in important implications for treatment and prognosis [20, 21]. After the use of NSAID in patients with endometrial cancer, the anti-inflammatory effects of the drugs could alter the immune environment of the tumor through the recruitment of different cytokines, thus affecting the mortality rate of patients with endometrial cancer [22, 23]. However, it was still unclear whether inflammation and its genes could affect the prognosis of endometrial cancer. In this study, we aimed to explore the prognostic role of an inflammation and its genes in endometrial cancer patients. A seven inflammation-related genes risk signature was conducted to predict the prognosis of patients with endometrial cancer by integrating high-throughput data. Our results showed that this prognostic model could accurately predict the prognosis of endometrial cancer, which may provide novel insights into clinical treatment of endometrial cancer.

## Methods

### Patient information and database

A total of 200 inflammatory-related genes (IRGs) were obtained from the gene set of HALLMARK_INFLAMMATORY_RESPONSE in the GSEA database (http://www.gsea-msigdb.org/) [24]. Clinical data, RNA-Seq, immune subtypes, and stemness scores based on DNA-methylation (DNAss) and mRNA (RNAss) were downloaded from the project TCGA-UCEC in the TCGA datasets (https://portal.gdc.cancer.gov/). Of all patient samples in TCGA-UCEC, 544 cancer samples and 53 para-cancerous samples met the requirement of corresponding complete age, gender, stage, overall survival (OS) and survival status, these qualified samples would be used for subsequent analysis. The RNA-seq data of GSE119041 and GSE21882 were obtained from the Gene Expression Omnibus (GEO) database (https://www.ncbi.nlm.nih.gov/geo/).

### Candidate prognostic inflammatory-related DEGs selection

Differentially expressed genes (DEGs) in cancer and adjacent tissues in the TCGA-UCEC project were screened by the “DEseq2” package in R software (R version 4.1.3) [25]. The screening conditions were: (*p* < 0.05; logFC filter > 1.5). Univariate Cox hazards regression analysis was performed on the obtained IRGs to generate candidate prognosis-related genes with a significant difference in OS (*p* < 0.05) by two-sided log-rank tests with the ‘survival’ package in R software.

### Construction and validation of IRGs-based risk assessment model

LASSO-COX univariate regression analysis was used to select potential prognostic factors based on candidate IRGs. Then, the Cox regression model was established with the “glmnet” package [26]. To measure the value of each IRGs in the risk assessment model, we calculated the regression coefficients in the univariate Cox hazards regression analysis. The formula for calculating risk score was listed as follows:$Risk score=\sum_{i=0}^{n}{Expr}_{i}*{Coef}_{i}$; ‘i’ for each IRG, including CCR7, GNA15, GPR132, LTA, MYC, NOD2, P2RX4, and P2RY2; ‘Expr’ for the gene expression level normalized by Log2; ‘Coef’ for the coefficient of IRG in the univariate Cox regression analysis. Patients were divided into 2 groups (high-risk and low-risk) according to the risk score, with the cutoff of the median risk scores. The Kaplan–Meier survival analysis were performed to analyze the prognostic difference between the two groups. By calculating the area under the time-dependent receiver operating characteristic (ROC) curve (AUC) with the “timeROC” package in R, the predictive power of IRGs was assessed [27]. Whether the survival status was well distributed in two risk groups was measured by both t-distributed stochastic neighbor embedding (t-SNE) and principal components analysis (PCA) mapping.

### Establishment and evaluation of prognostic nomogram

The IRGs were selected to be an independent prognostic factor for patients with endometrial carcinoma by univariate Cox regression analysis in the TCGA-UCEC data. These IRGs were integrated to establish a genomic nomogram for predicting the 1-year, 2-year, and 3-year survival possibility of each patient. The score for each IRG was summed by the formula listed above. The nomogram incorporating IRGs for predicting overall survival was plotted with the ‘rms’, ‘nomogramEx’, and ‘regplot’ package. To determine whether the risk score calculated based on IRGs was a significant predictor of prognosis along with other potential risk factors such as age, grade, lymphatic metastasis, and stage, univariate and multivariate Cox Hazards regression analysis were performed.

### Tumor microenvironment characteristics analysis

Single-sample gene set enrichment analysis (ssGSEA) was conducted for quantifying the immune-related pathway scores and immune cell scores in two different groups. The relative R package was “GSVA”. Besides, the stromal score and immune score for each patient were calculated using the ESTIMATE function of the R package.

### Drug sensitivity anaylsis

The correlation between the expression of IRGs and the sensitivity of chemotherapy drugs was quantified using the CellMiner tool (https://discover.nci.nih.gov/cellminer). This database contained 60 different cell lines which must be screened when developing new anti-tumor drugs and 262 drugs licensed by FDA or on clinical trials.

### Protein–protein interaction (PPI) analysis and enrichment assays

Protein–protein interaction (PPI) data was obtained from the Search Tool for Retrieval of Interacting Genes/Proteins (STRING) database (http://string-db.org/). The interaction network was constructed based on the IRGs with the species limited to “homo sapiens” and the setting confidence > 0.7 [28]. IRGs were subjected to the Kyoto Gene and Genomic Encyclopaedia (KEGG) pathway by R software. *p* < 0.05 was considered as a significant difference.

### Verification of the mRNA expression and biological function of IRGs

We collected 15 endometrial cancer tissuses and 10 normal endometrial tissues and the total RNAs were isolated by Trizol reagent (TaKaRa, Japan) for gene expression detection. After that, RT-PCR measurement was carried out on the StepOnePlus Real-Time PCR system (Applied Biosystems, USA). β-Actin was used as an endogenous reference gene. In order to explore the effect of IRGs on tumor growth, small interference RNA transfection experiment was conducted in the human endometrial cancer cell line of RL-952 and HEC-1B. Cell lines were cultured in complete high glucose Dulbecco’s modified Eagle medium (DMEM, China) with 10% fetal bovine serum and 100 μg/ml penicillin–streptomycin (Hyclone, USA). Cells were incubated at 37 °C with 5% CO2 incubator. After seeded in a 6-well plate (5*10^5^/well), cells were transfected with P2RY2 siRNA (GenePharma, China) and control siRNA with Lipofectamine® 2000 transfection agent (Invitrogen, USA). Cell viability was measured by CCK8-kit (Dojindo, Japan). The cells were seeded in 96-well plates with a density of 3000 cells per well at 37 °C overnight, 100 μl per well of CCK8 was then added. The activity of the cells was determined at 450 nm absorbance after being incubated for 2 h.

### Statistical analysis

All statistical analyses were performed using R software (R version 4.1.3) and GraphPad Prism 7. *p* < 0.05 was considered to be significantly different. All gene expression data were Log2 corrected. Multivariate as well as Univariate Cox proportional hazards analysis was performed to evaluate the hazard ratios of relevant variables. Kaplan‐Meier survival curves were plotted to compare the survival difference between high- and low-risk groups. The ROC curves were used to show the accuracy of the prognostic prediction model. One-way ANOVA was adopted to calculate the difference in the specific characteristics between high- and low-risk groups.

## Results

### Inflammation-related genes in endometrial carcinoma

**Fig. 1:**
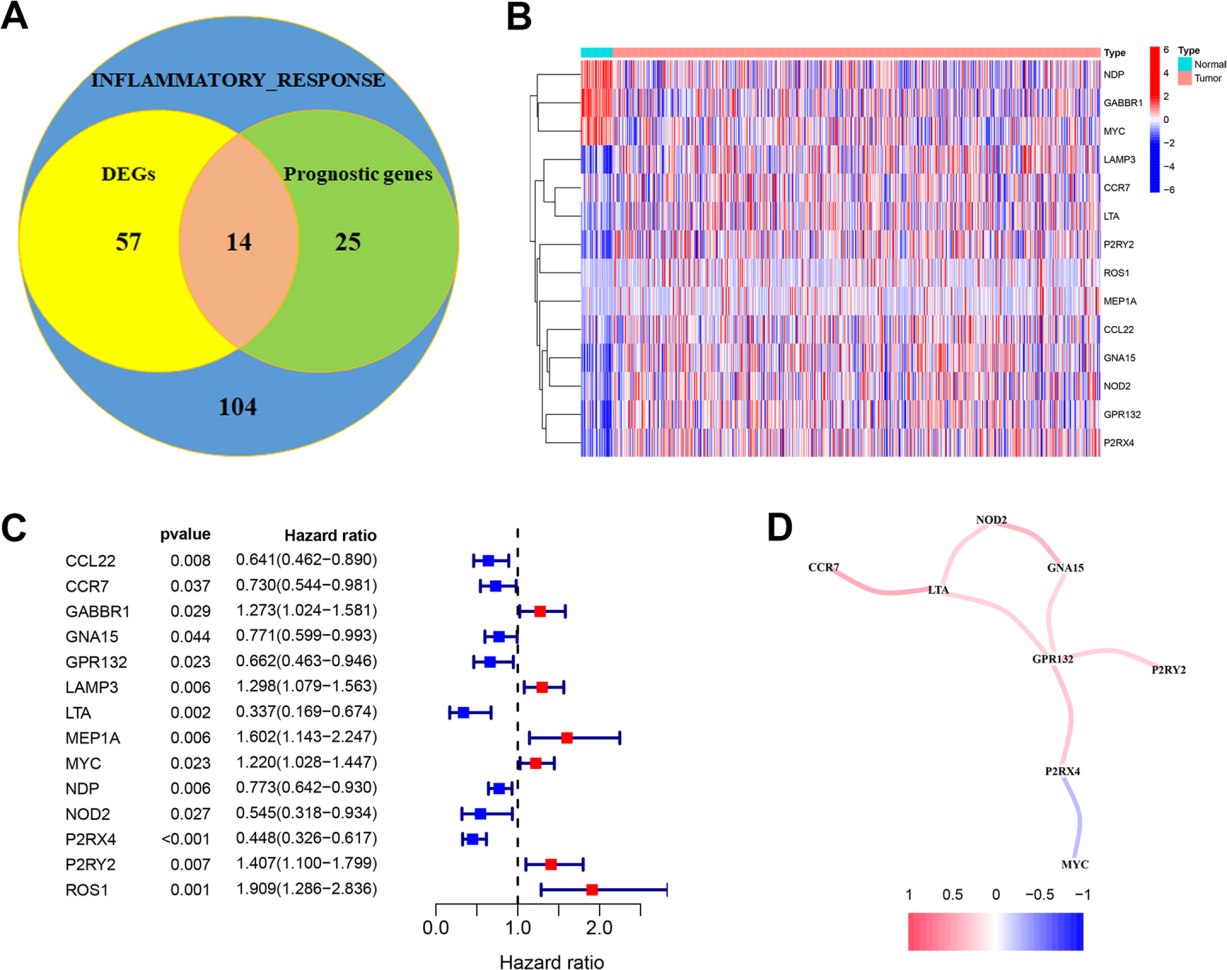
Inflammation-related genes in endometrial carcinoma. **A** Venn diagram showing the 14 overlapped genes of DEGs and prognostic genes correlated with the inflammatory response. **B** Expression heatmap of signature genes in cancer and para-cancerous group. **C** Forest map of hazard ratios for signature genes. **D** Correlation analysis of signature genes. Red for positive correlation; Blue for negative correlation

To construct a prognosis prediction model in endometrial carcinoma, we analyzed 200 inflammation-related genes (IRGs) in 544 endometrial carcinoma samples and 53 para-cancerous samples from TCGA database. 71 differential expression genes were identified using t-test in R (|log2FC|> 0.5, *p* < 0.05). Univariate Cox regression of all the IRGs showed that there have been 39 IRGs which had significant prognostic values in endometrial carcinoma overall survival. Fourteen overlapped genes were shown in the Venn diagram (Fig. 1A). The expression of 11 up-regulated genes (LAMP3, CCR7, LTA, P2RY2, ROS1, MEP1A, CCL22, GNA15, NOD2, GPR132, P2RX4) and 3 downregulated genes (NDP, GABBR1, MYC) were visualized in the heatmap (Fig. 1B). Six of the 14 IRGs were considered as high risk factors, while the remaining 8 IRGs indicated better survival in endometrial carcinoma patients (Fig. 1C). Pearson's correlation analysis calculated the correlation network between the signature genes (Fig. 1D). Nearly all of the prognostic signature genes were positively correlated, but the relationship between P2RX4 and MYC was negative.

### Construction and verification of a prognostic model for endometrial carcinoma patients

**Fig. 2:**
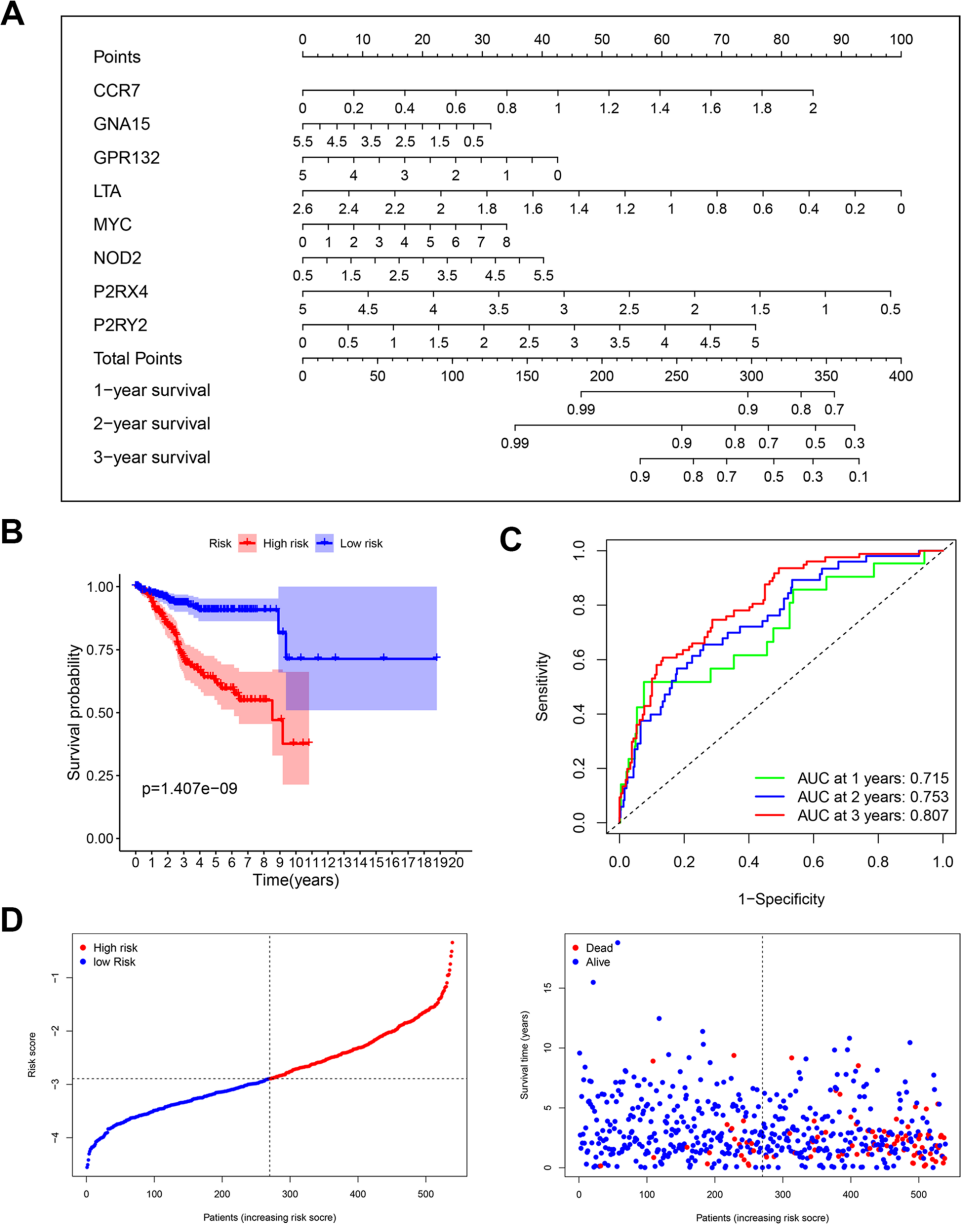
Construction and verification of a prognostic model for endometrial carcinoma patients. **A** Nomogram for predicting 1-year, 2-year, and 3-year overall survival in endometrial carcinoma patients. **B** Kaplan–Meier curves for overall survival according to the nomogram. **C** ROC of 1-year, 2-year, and 3-year survival according to the nomogram. **D** The distribution diagram of survival status along with risk score;

We aimed to establish an available inflammation-related gene model (IRGSM), which could predict prognosis by evaluating the inflammation-related signatures. Eight IRGs were incorporated to construct a nomogram for survival prediction in endometrial carcinoma patients, including CCR7, GNA15, GPR132, LTA, MYC, NOD2, P2RX4 and P2RY2 (Fig. 2A). The relative 1-year, 2-year, and 3-year survival rates were determined after a straight-line drawing from the added points for each predictor gene on the total point axis to the survival probability axis. Patients were subclassified into low- or high-risk groups based on the median risk score. Kaplan–Meier curves showed a significant difference in survival probability between the high- and low-risk groups (Fig. 2B). Time-dependent receiver operating characteristic (ROC) analysis showed satisfied sensitivity of IRGSM to predict prognosis with the area under the curve (AUC) over 0.7 for 1-year, 2-year, and 3-year survival probability (Fig. 2C). In order to assess whether the risk score could well stratify patients, we plotted the distribution of patient survival status and risk score (Fig. 2D). Patients in the low-risk group (on the left side of the dotted line) had more living and longer survival time than the high-risk group (on the right side of the dotted line).

### Prognostic value of the candidate prognostic model

**Fig. 3:**
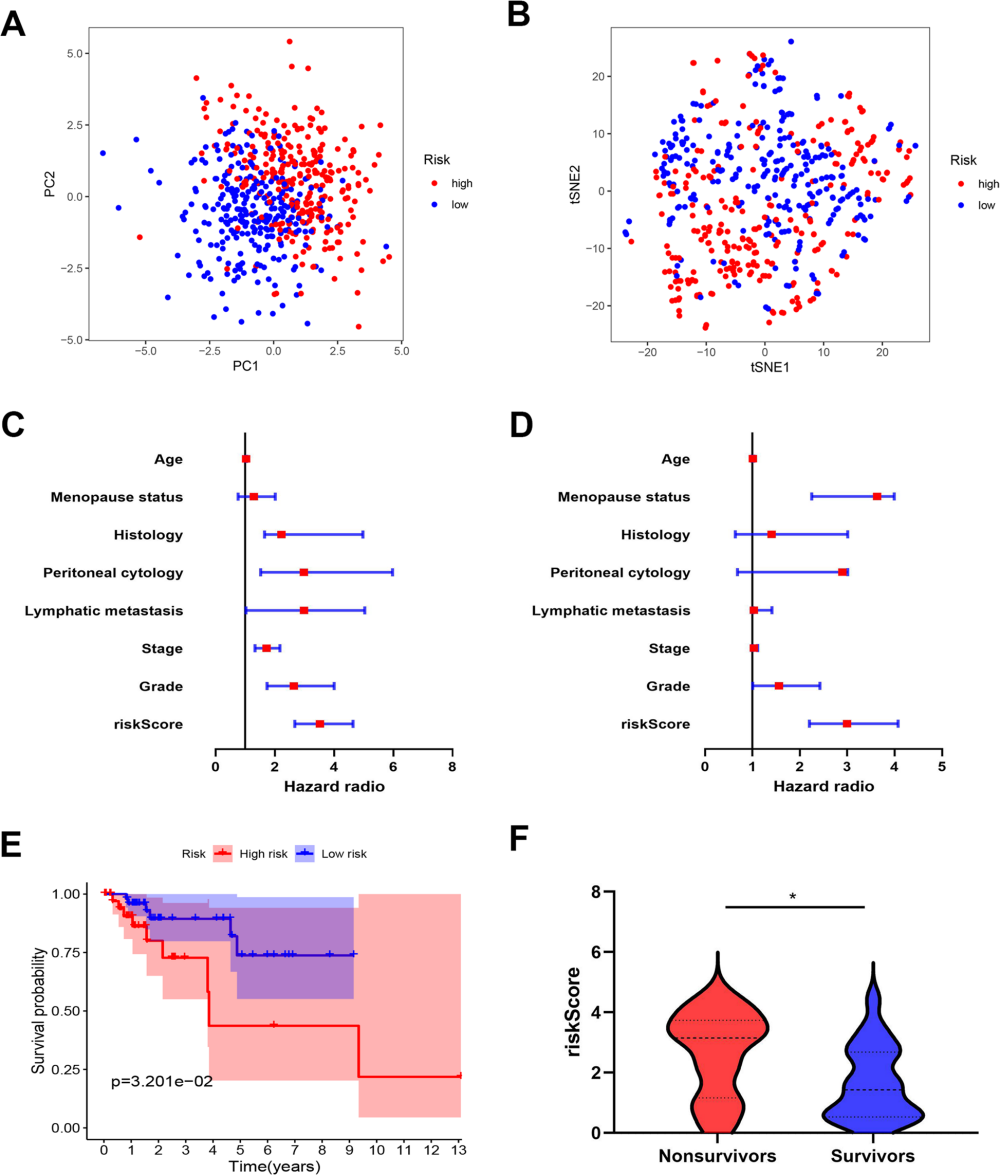
Prognostic value of the candidate prognostic model. **A** PCA plot for low- and high-risk endometrial carcinoma patients. **B** tSNE plot for low- and high-risk endometrial carcinoma patients. **C** Univariate COX regression analysis for the TCGA cohort. **D** Multivariate COX regression analysis for the TCGA cohort. **E** Kaplan–Meier curves produced survival analysis of GSE119041. **F** 5-year survival status in GSE21882

After patients were divided into low- and high-risk groups according to the risk scores, both principal component analysis (PCA) and t-distributed stochastic neighbor embedding (t-SNE) were utilized to visualize the distribution of patients (Fig. 3A-B). To further evaluate whether the risk factor calculated by the gene signatures had an independent prognostic predicting function, univariate and multivariate Cox regression analysis was performed. The results showed that the hazard ratio of risk score was 2.995, 95%CI: 2.201 ~ 4.074 by univariate analysis (*p* < 0.05) and 3.525, 95% CI: 2.678 ~ 4.640 by multivariate analysis (*p* < 0.05) (Fig. 3C-D). Additionally, to further confirm the reliability of the IRGSM, GSE119041 and GSE21882 from GEO databases were selected as validation cohorts. As shown in Fig. 3E-F, prognosis was significantly worse in the high-risk group compared with the low-risk group. These results indicated that our model performed robustly in different cohorts.

### Tumor microenvironment analysis based on IRGSM

**Fig. 4:**
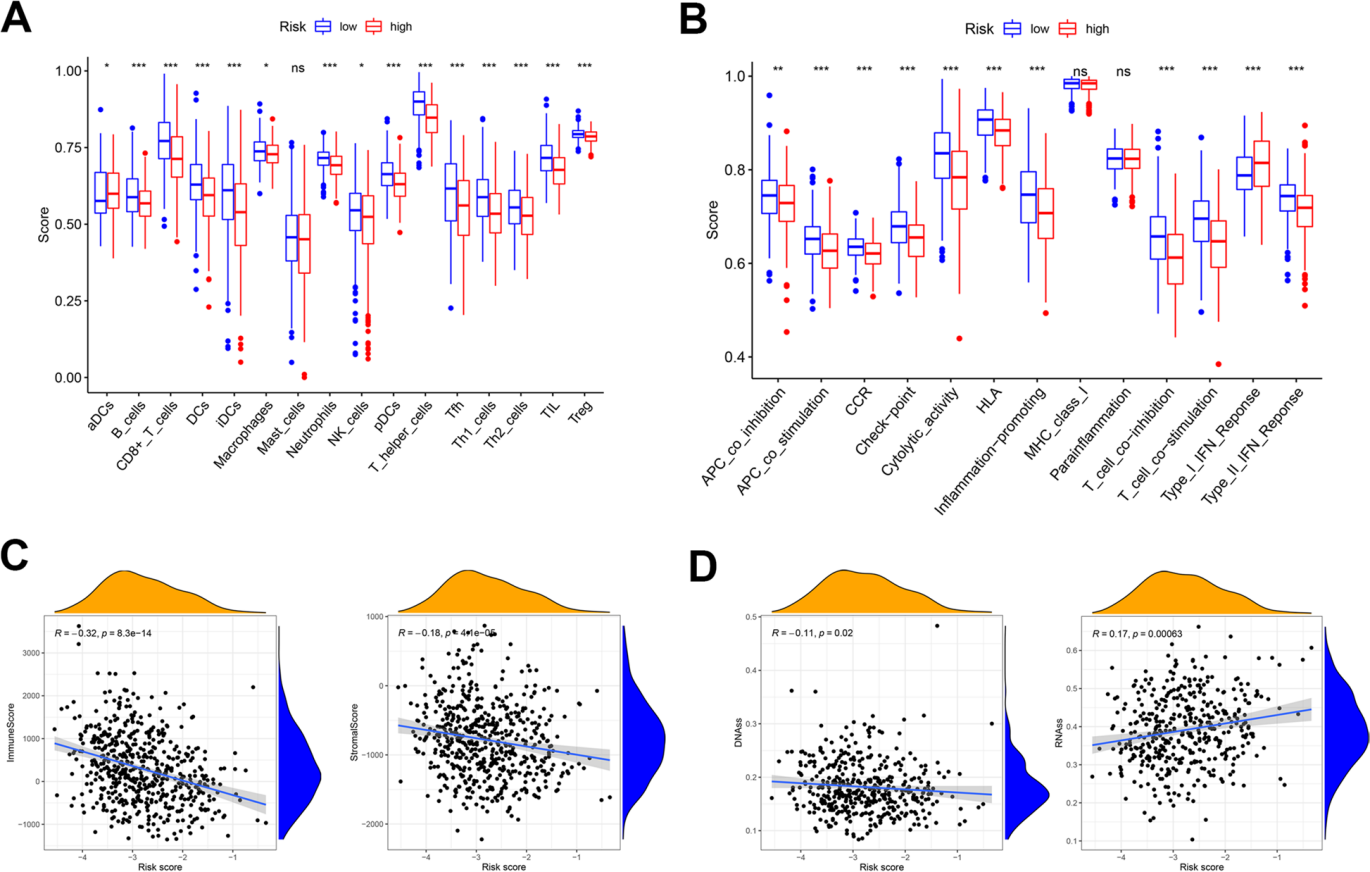
Tumor microenvironment analysis based on inflammatory-related risk scores. **A** Box plot showing the enrichment scores of 16 immune cells in low-(blue) and high-risk groups (red). **B** Box plot showing the enrichment scores of 13 immune-related pathways between two groups. **C** Correlation analysis between risk score and immune score, and stromal score. **D** Correlation analysis between risk score and DNAss, RNAss

To explore the impact of prognostic IRG signatures on immune activity, we compared the enrichment and correlated properties of immune cells and immune-related pathways between the low- and high-risk groups by applying single-sample gene set enrichment analysis (ssGSEA) in the TCGA cohort (Fig. 4A-B). The high-risk group was characterized by a generally low immune cell infiltration status. The results for the immune-related pathway were consensus with the immune cells, as most of the pathway were downregulated in the high-risk group except for type I_IFN_response. The above analysis indicated that the immune response in endothelium carcinoma might result in an optimistic outcome. The stemness score based on mRNA expression (RNAss) and DNA methylation pattern (DNAss) were analyzed to determine the tumor stemness (Fig. 4C). The results showed a positive correlation between RNAss and risk score (*r* = 0.17, *p* < 0.05). Immune status and stromal cells were major components of the tumor microenvironment, both of which were negatively associated with risk scores (*p* < 0.05) (Fig. 4D).

### Functional analysis of IRGs

**Fig. 5:**
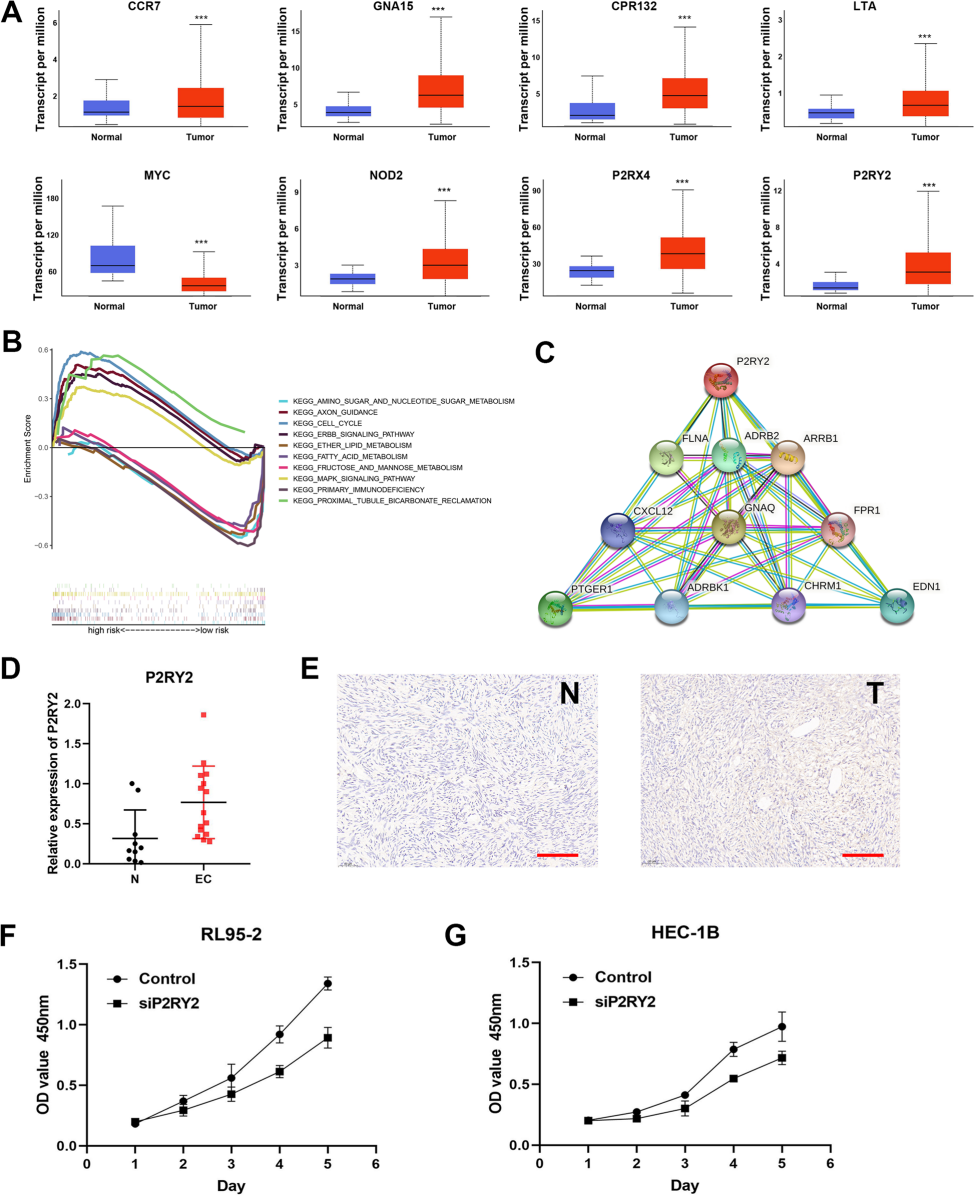
Exploration and validation of the function of IRGs in endometrial cancer. **A** Boxplot showing the expression level of IRGs in TCGA. **B** KEGG analysis showing functional enrichment in risk groups. **C** PPI network showing the correlation between IRGs proteins. **D**-**E** P2RY2 expression in normal and endometrial carcinoma tissuses (scale bar = 100 μm). **F** Cell viability of siP2RY2 and control group in RL95-2 and HEC-1B cell lines

To further explore the function of IRGs in endometrial cancer, the differences in the expression of IRGs in endometrial cancer tumor tissues and adjacent tissues were analyzed based on TCGA database (Fig. 5A). The expression of risk factor MYC was decreased in tumors, while the other seven IRGs were highly expressed in tumors (*P* < 0.05). To investigate the intrinsic function changes in patients of two different risk groups, the KEGG analysis was performed (Fig. 5B). Top five pathways were enriched in the high-risk group, encompassing the cell cycle, ERBB signaling pathway, and MAPK signaling pathway. On the contrary, another top five pathways associated with mannose, lipid, and nucleotide sugar metabolic process were enriched in the low-risk group. The interaction between IRGs proteins was explored using STRING (Fig. 5C). Proteins with significant associations were mapped in the PPI network. The genes that were physically and functionally closely related to IRGs included P2RY2, FLNA, ADRB2, ARRB1, CXCL12, GNAQ, FPR1, PTGER1, ASRBK1, CHRM1, and EDN1. Finally, we validated the expression and function of IRGs in endometrial cancer samples and cell lines (Fig. 5D). Taking P2RY2 as an example, RT-PCR results showed that the expression level in the tumor was significantly higher than that in the adjacent tumor. In the immunohistochemical staining results of endometrial cancer tissues, we also found that the expression of P2RY2 was higher in tumor tissues (Fig. 5E). In the RL95-2 and HEC-1B cell lines, cell growth was significantly decreased after the knockdown of P2RY2 which was demonstrated by CCK-8 assay (Fig. 5F). The result suggested that P2RY2 could promote tumor proliferation, which was consistent with our previous inference on the role of IRGs in endometrial cancer.

### The correlation between IRGs and drug sensitivity

**Fig. 6:**
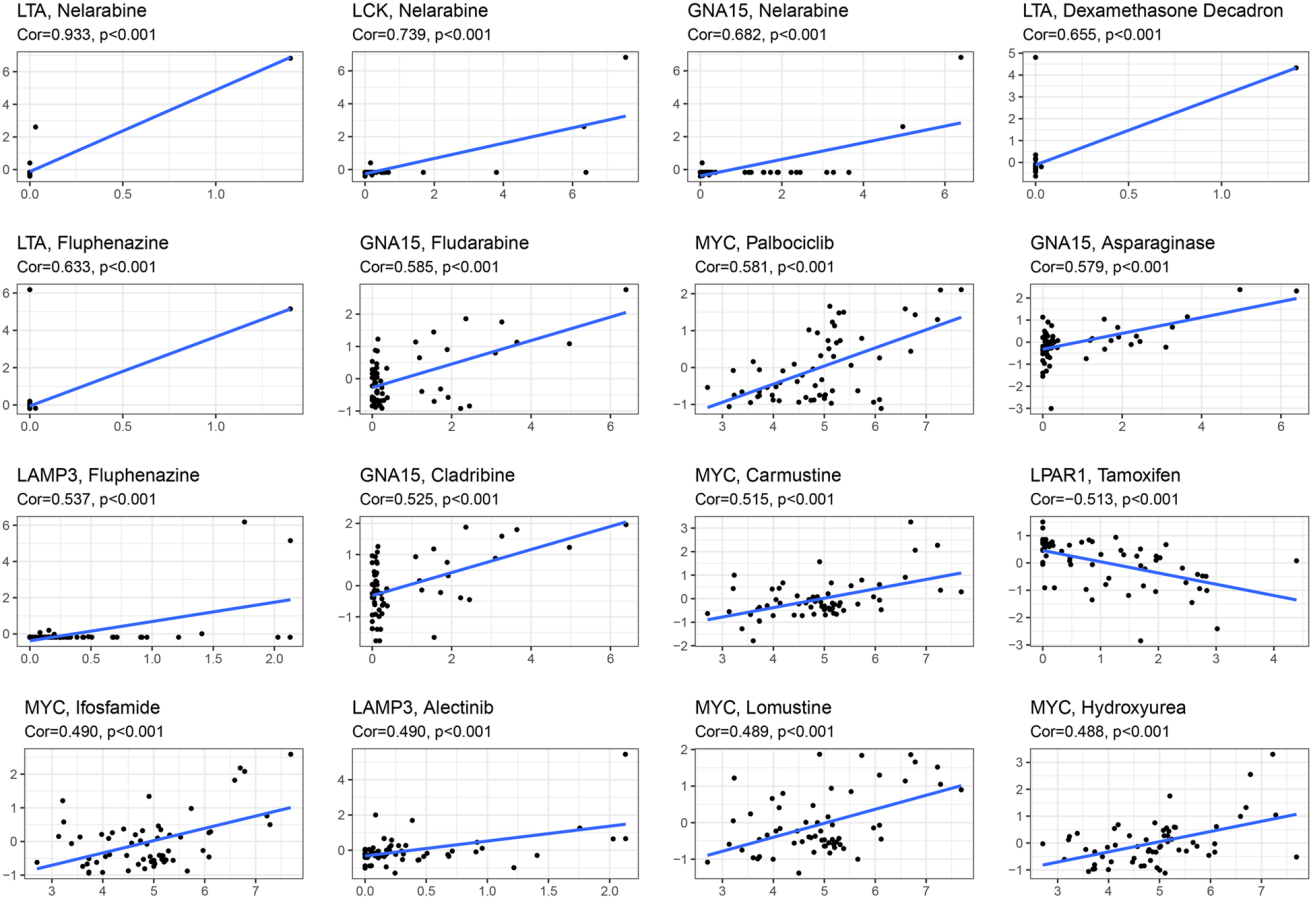
The correlation between IRGs and drug sensitivity

To explore the clinical application utility of the IRGSM, we predicted cancer cell sensitivity to anti-tumor drugs (Fig. 6). Most of the signature genes enhanced the drug response of cancer cells (*P* < 0.01). For instance, the upregulation of GNA15, LTA, LAMP3, LCK, and MYC was associated with increased cell sensitivity including Cladribine, Asparaginase, Nelarabine, Fludarabine, Nelarabine, Fluphenazine, Alectinib, Lomustine, Hydroxyurea, Ifosfamide. However, LPAR1 increased drug resistance to Tamoxifen (*P* < 0.01).

## Discussion

The high ability of tumor cells to invade and metastasize results in a low 5-year survival rate for patients with endometrial cancer [29, 30]. In recent years, studies on prognostic risk prediction models for endometrial cancer have gradually increased [31, 32]. Inflammation has been found to play a role in endometrial cancer, but only a few studies have developed inflammation-based prognostic markers [33]. An eight inflammation-related genes prognosis signature for endometrial cancer precisely identified the survival of endometrial cancer patients in robustness evaluation. Inflammation-related genes could serve as possible biomarkers and potential therapeutic targets for patients with endometrial cancer.

It is a very effective bioinformatics strategy to establish predictive models using data from TCGA and GEO databases that sequenced the whole genome of endometrial cancer patients. In recent years, increasing attention has been paid to the gene characteristics related to the inflammatory. A previous study has demonstrated that eight inflammatory response-related genes can be used for prognostic prediction and impact the immune status in hepatocellular carcinoma and suppressing these genes may be a treatment option [34]. However, the prognostic model of inflammation-related genes in endometrial cancer has not been reported yet. Hence, our study integrated the data from the TCGA and GEO databases and finally identified eight IRGs, including CCR7, GNA15, GPR132, LTA, MYC, NOD2, P2RX4 and P2RY2. Among these genes, CCR7, MYC and NOD2 have been reported in a large number of tumor studies, including endometrial cancer, while P2RX4, GNA15, and GPR132 genes have few molecular biological experiments to verify their role in endometrial cancer progress [35–38]. P2RY2 is G protein coupled purinergic receptors that induce a signaling cascade through different second messengers [39]. Recent studies in different models of physiological processes have demonstrated the participation of the P2RY2 receptor in inducing migration or the epithelial to mesenchymal cell transition (EMT) process [40]. Thus, we verified the expression of P2RY2 in endometrial cancer tissues and its effect on the growth of endometrial cancer cells primarily. The in vitro results were also consistent with the above bioinformatics analysis.

Inflammatory mediators and cellular effectors are important components of the local tumor environment. In some types of cancer, including hepatocellular carcinoma, inflammation occurs prior to the onset of malignant changes [41, 42]. In contrast, in other types of cancer, tumors could alter the inflammation-inducing microenvironment and promote tumor development [43, 44]. Regardless of its origin, tumor progression is tied to inflammation in the tumor microenvironment. Inflammation contributes to tumor cell proliferation and survival, promotes tumor angiogenesis and metastasis, disrupts adaptive immune responses, and alters the response of tumor lesions to chemotherapeutic agents. However, there are fewer studies on the relationship between endometrial cancer and inflammation. Our study evaluated the ability of inflammation-associated genes to predict the prognosis of endometrial cancer patients and also to construct a prognostic prediction model based on inflammation-associated genes.

In this study, we established an inflammatory risk model to predict the prognosis of endometrial cancer based on TCGA database. Firstly, differentially expressed inflammatory genes were identified and constructed to a prognostic model by means of LASSO. The bioinformatic analysis, including ROC, risk score, Kaplan Meier analysis, univariate and multivariate cox regression analysis, proved the excellent ability to predict prognosis of the gene signatures based on inflammation. Finally, we performed tumor microenvironment characteristics analysis and drug sensitivity analysis of these differentially expressed genes. In summary, higher risk score was found to be strongly associated with poorer prognosis of endometrial cancer, which could effectively help clinicians making accurate and effective decisions.

## Data Availability

The data that support the findings of this study are available on request from the corresponding author.
